# Calibration of Laser Beam Direction for Inner Diameter Measuring Device

**DOI:** 10.3390/s17020294

**Published:** 2017-02-05

**Authors:** Tongyu Yang, Zhong Wang, Zhengang Wu, Xingqiang Li, Lei Wang, Changjie Liu

**Affiliations:** State Key Laboratory of Precision Measuring Technology and Instruments, Tianjin University, Tianjin 300072, China; yangtongyu_tju@163.com (T.Y.); wangzhong@tju.edu.cn (Z.W.); wuzhengang@tju.edu.cn (Z.W.); lxq.792751045@163.com (X.L.); wanglei2014@tju.edu.cn (L.W.)

**Keywords:** inner diameter measurement, laser beams, direction calibration, laser displacement sensor

## Abstract

The laser triangulation method is one of the most advanced methods for large inner diameter measurement. Our research group proposed a kind of inner diameter measuring device that is principally composed of three laser displacement sensors known to be fixed in the same plane measurement position. It is necessary to calibrate the direction of the laser beams that are emitted by laser displacement sensors because they do not meet the theoretical model accurately. For the purpose of calibrating the direction of laser beams, a calibration method and mathematical model were proposed. The inner diameter measuring device is equipped with the spindle of the machine tool. The laser beams rotate and translate in the plane and constitute the rotary rays which are driven to scan the inner surface of the ring gauge. The direction calibration of the laser beams can be completed by the sensors’ distance information and corresponding data processing method. The corresponding error sources are analyzed and the validity of the method is verified. After the calibration, the measurement error of the inner diameter measuring device reduced from ±25 μm to ±15 μm and the relative error was not more than 0.011%.

## 1. Introduction

Efficient and accurate measurement of the key parameters has always been an important subject in field of manufacturing. Especially for the workpieces with a large round hole whose diameter is bigger than 250 mm, such as vehicles, marine engine cases and large gas turbine impellers, the measuring accuracy of the inner diameter directly determines the assemble efficiency and products’ quality [1]. Inner dimensional inspection is traditionally performed by micrometer, caliper gauge, and other telescopic mechanical tools [2,3]. “Large size” measurement traditionally referred to a measurement size exceeding 500 mm, but with the improvement of measurement accuracy requirements, “large size” measurement refers to a measurement size exceeding 250 mm [4]. The coordinate measuring machine (CMM) can complete the large size measurement tasks because of its high measurement accuracy and advanced technology [5,6,7]. However, the costs and measurement efficiency restricts the wide use of CMM [8]. To reduce the costs and time, and to increase measurement efficiency for dimensional inspections, researchers and engineers have been exploring a series in situ measuring methods in recent years [9]. To meet these requirements, non-contact measurement technology has become the research focus of industrial inspection. The main non-contact measurement methods include visual detection method [10,11] and laser scanning method [12], etc. According to the principle of a circumscribed circle of a triangle which is formed by three points not on the same straight line, our research group developed an inner diameter measuring device with three-laser displacement sensors; it is mainly through these three-laser displacement sensors scanning the hole that the task is completed, so the laser beam direction has to be calibrated before measurement. Bi et al. developed a method to calibrate the laser beam direction by the use of CMM and spherical target [13], this method depended on the CMM and special spherical target, limiting it from wide use. Sun et al. used CCD (Charge Coupled Device) cameras to calibrate the laser beam direction [14]. This method can only calibrate one beam direction at a time. Xie et al completed the calibration task through a planar target etched with grid lines to generate calibration points [15], the measurement range of this method was small and there were certain restrictions on the use of the environment. Lee and Shiou proposed a method with five laser beams to measure complex curved surface [16], but it is not suitable for calibration of the laser beams’ direction with too large of an angle. Kai et al. proposed an algorithm to calibrate the laser beam direction with CCD camera in a small field of view [17,18]. Yu et al. used the grating scale to calibrate [19], but this method was not suitable for industrial applications, because the grating scale needs auxiliary equipment to complete the measurement and calibration tasks and the system could become more complex and expensive. Due to the influence of various factors in the industrial field, the accuracy of high precision grating scale is affected too. The general problem in the methods of spot laser beam direction calibration is their small range, strict environmental restrictions and only one laser beam can be calibrated [20,21,22,23]. To meet the requirements of large-size diameter measurement, this paper proposes an actual mathematical model of the inner diameter measurement device and calibrated three laser beams’ direction through CNC (Computerized Numerical Control) machine tools and standard ring gauge. The main contributions of this paper are to calibrate the direction of three laser beams simultaneously and realize the in situ calibration of the laser beam direction. After the calibration, the inner diameter measuring device can independently measure and abandon the auxiliary of CNC. 

## 2. Calibration Model

### 2.1. The Measurement Principle and Theoretical Mathematical Model 

**Figure 1:**
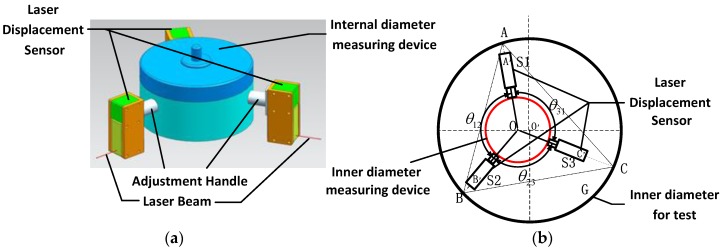
(**a**) Physical model; (**b**) Theoretical model.

Our research group developed a kind of inner diameter measuring device to measure the inner diameter of large size parts whose diameter is bigger than 250 mm. The measuring range of the leaser displacement sensors cannot be too large, because the measurement accuracy has to be maintained. Our research group had proposed a method to solve this problem by increasing the number of sensors [24], but this method might cause the measuring device volume to be too large and decrease the generality of the device. In order to increase the measuring range with a lower sensors number, this paper proposes a new kind of device. The physical model is shown in Figure 1a, three-laser displacement sensors are installed on the adjustment handle. Adjusting the handle can measure different kinds of parts by only three sensors and increase measurement range. The new theoretical model and calibration method were built, followed by the new physical model. The theoretical model is shown in Figure 1b. The inner diameter measurement device mainly consists of three laser displacement sensors for which the location is known.

In the theoretical model image, *G* is the hole whose inner diameter is the key parameter that we need to measure. Point $O^{\prime }$ is the center of *G* and point O is the center of the measuring device. The measuring device is placed in *G*, its three laser displacement sensors *S*1, *S*2 and *S*3 are equally distributed in the circumferential direction at $\theta_{12}$, $\theta_{23}$, $\theta_{31}$ and their laser beams converge at the point *O*. $A^{\prime }$, $B^{\prime }$ and $C^{\prime }$ are the measurement starting points of the three laser displacement sensors, and $A^{\prime }A$, $B^{\prime }B$ and $C^{\prime }C$ are the measuring value of the sensors. *G* is the circumcircle of triangle *ABC*, as shown in Figure 1, in which $OA=OA^{\prime }+A^{\prime }A$, $OB=OB^{\prime }+B^{\prime }B$, $OC=OC^{\prime }+C^{\prime }C$. Each side of triangle *ABC* can be expressed as below according the cosine theorem:

(1) $$\left\{ \begin{array}{l} AB=\sqrt{OA^{2}\times OB^{2}-2OA\times OB\times \cos\theta_{12}}\\ BC=\sqrt{OB^{2}\times OC^{2}-2OB\times OC\times \cos\theta_{23}}\\ AC=\sqrt{OA^{2}\times OC^{2}-2OA\times OC\times \cos\theta_{31}} \end{array}\right.$$

The area *S* of the triangle *ABC* can be written as:

(2) $$S=\frac{1}{4}\sqrt{\left(AB+AC+BC\right)\left(-AB+AC+BC\right)\left(AB-AC+BC\right)\left(AB+AC-BC\right)}$$

As shown in Figure 1, the hole *G* is the circumcircle of triangle *ABC*. Hence, the diameter of the hole *G* is equal to two times the radius, and the radius *R* can be expressed as below:

(3) $$R=\frac{AB\times BC\times AC}{4S}$$

Due to the processing, material and other factors, the laser beams cannot accurately fulfil the theoretical model. The measurement experiment of the standard ring gauge had shown that the error of the inner diameter measuring device was 25 μm based on theoretical model. So, it was necessary to build an actual model of the measurement device and calibrate the direction of three laser beams.

### 2.2. The Actual Mathematical Model

**Figure 2:**
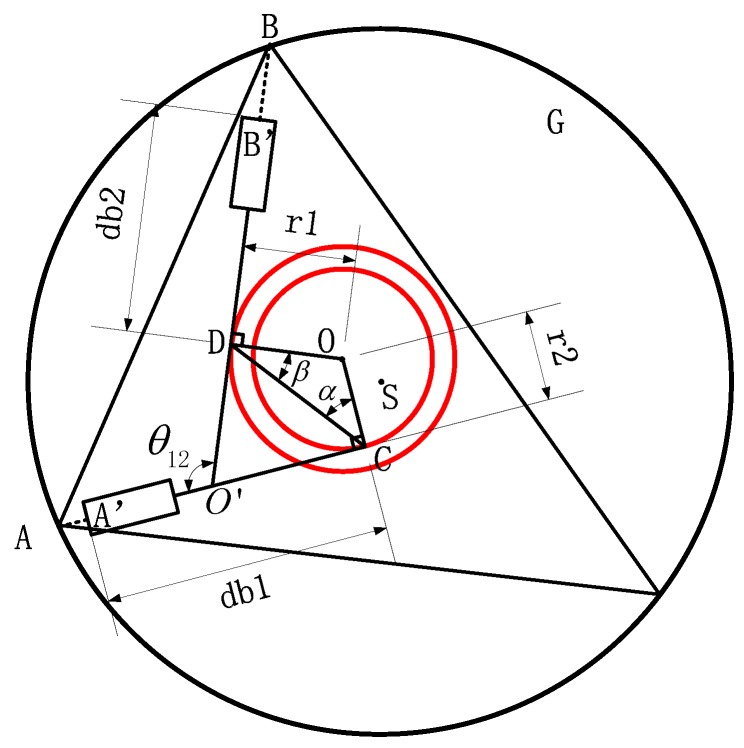
The actual mathematical model.

It is necessary to know the constraint parameters of the laser beam before the calibration of direction. The three laser beams are in the same plane and cannot converge at one point; when the measuring device rotates and scans the hole, three laser beams wrap three concentric circles around the center of rotation and the laser beam tangents to the corresponding concentric circle. The actual mathematical model is shown in Figure 2.

As shown in Figure 1b, during the rotation scanning process, the laser beams $A^{\prime }A$, $B^{\prime }B$ and $C^{\prime }C$ could envelope three concentric tangential circles with O as the rotation center, and *S* as the center of the hole *G*. As shown in Figure 2, taking the line *AB* of the measuring points *A* and *B* as an example, the laser beams $A^{\prime }A$ and $B^{\prime }B$ instersect at the point $O^{\prime }$ and the angle between $A^{\prime }A$ and $B^{\prime }B$ is $\theta_{12}$; point $A^{\prime }$ and $B^{\prime }$ are the measuring starting points of the laser displacement sensors, points *C* and *D* are the cut-off points of laser beams to the tangential circles, and the radius of the tangential circles are labelled as follows: the tangential circles *r*, $r_{1}$ and $r_{2}$ denote *OD* and *OC* are the tangential circles’ radius; the distance form the measuring starting points to cut-off points are called as base circle radius $d_{b}$, such as $d_{b1}$ and $d_{b2}$ in Figure 2; ∠OCD=α and ∠ODC=β. According to the cosine theorem, the side *AB* of triangle *ABC* can be expressed as:

(4) $$\left\{ \begin{array}{l} AB=\sqrt{O^{\prime }A^{2}+O^{\prime }B^{2}-2\times O^{\prime }A\times O^{\prime }B\times \cos\theta_{12}}\\ O^{\prime }A=d_{b1}+A^{\prime }A-O^{\prime }C\\ O^{\prime }B=d_{b2}+B^{\prime }B+O^{\prime }D \end{array}\right.$$

In the quadrilateral $ODO^{\prime }C$, *CD* can be expressed as:

(5) $$CD=\sqrt{{r_{1}}^{2}+{r_{2}}^{2}-2\times r_{1}\times r_{2}\times \cos\theta_{12}}$$

In the triangle *ODC*, α and β can be expressed as:

(6) $$\left\{ \begin{array}{l} \alpha =\arcsin\left(r_{1}\frac{\sin\theta_{12}}{CD}\right)\\ \beta =\arcsin\left(r_{2}\frac{\sin\theta_{12}}{CD}\right) \end{array}\right.$$

In triangle $O^{\prime }DC$ the line segment *CD* can be expressed as:

(7) $$\left\{ \begin{array}{l} O^{\prime }D=CD\frac{\sin\left(\frac{\pi }{2}-\alpha \right)}{\sin\left(\pi -\theta_{12}\right)}\\ O^{\prime }C=CD\frac{\sin\left(\frac{\pi }{2}-\beta \right)}{\sin\left(\pi -\theta_{12}\right)} \end{array}\right.$$

Taking Equations (4)–(7) into Equation (3), Equation (3) can be deduced as below:

$$R=f\left(A^{\prime }A,B^{\prime }B,C^{\prime }C,r_{1},r_{2},r_{3},\theta_{12},\theta_{13},\theta_{23},d_{b1},d_{b2},d_{b3}\right)$$

$A^{\prime }A$, $B^{\prime }B$ and $C^{\prime }C$ are the sensors’ measured values, so the constraint parameters of the laser beams are the radius *r* of tangential circles, radius $d_{b}$ of base circles and the angle θ between two adjacent laser beams.

### 2.3. The Calibration Principle

**Figure 3:**
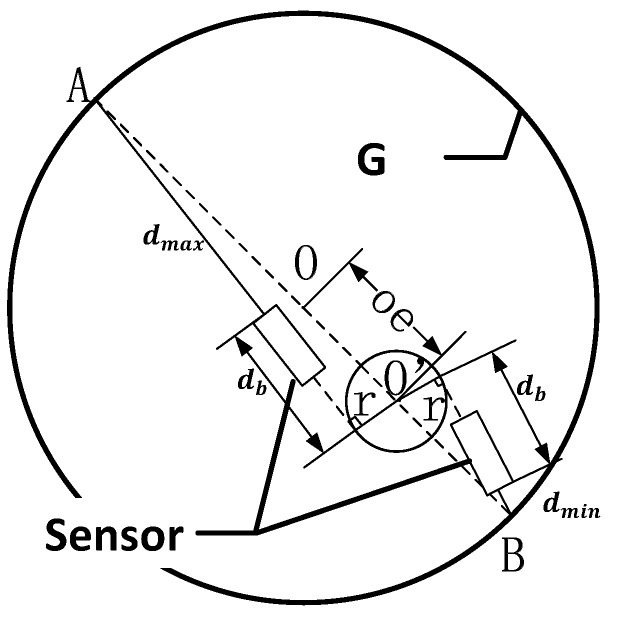
The calibration principle of tangential circles radius *r* and base circles radius $d_{b}$.

The calibration principle is divided into two parts, the first part is to calibrate the radius of tangential circles and base circles, and the second part is to calibrate the angle between two adjacent beams. Since the calibration method of each laser beam’s base circle radius *r* and tangential circle radius $d_{b}$ are the same and independent, this paper is only proposed the method for how to calibrate one laser displacement sensor’s laser beam. The calibration principle about how to calibrate the tangential circles radius *r* and base circles radius $d_{b}$ is shown in Figure 3.

In Figure 3, *G* is the hole to be measured and the point *O* is the center of the hole. In the calibration process, the measuring device is equipped with the machine tool spindle and rotary scans with the standard ring gauge whose radius is known. As shown in Figure 3, a single sensor rotation center $O^{\prime }$ does not coincide with the center point *O* and the eccentricity between $O^{\prime }$ and *O* is *oe* [25]. The laser displacement sensor rotates around point $O^{\prime }$ to scan the hole, at point *A* the sensor obtains the maximum measuring value and at point *B* the sensor obtains the minimum measuring value. Because the r and $d_{b}$ are fixed values and point *A*, point *O*, point $O^{\prime }$ and point *B* are in the same diameter, so Equation (8) can be expressed as below:

(8) $$\left\{ \begin{array}{l} {\left(d_{b}+d_{\min}\right)}^{2}+r^{2}={\left(R-oe\right)}^{2}\\ {\left(d_{b}+d_{\max}\right)}^{2}+r^{2}={\left(R+oe\right)}^{2} \end{array}\right.$$

where *R* is obtained by the standard ring gauge. The coordinate $\left(x_{0},y_{0}\right)$ of point O is obtained by a hole center positioning method which was proposed by Wang [26] and the coordinate $\left(x_{1},y_{1}\right)$ of the point $O^{\prime }$ is obtained by the machine tool, so the value of *oe* can be expressed as below:

(9) $$oe=\sqrt{{\left(x_{1}-x_{0}\right)}^{2}+{\left(y_{1}-y_{0}\right)}^{2}}$$

Taking Equation (9) into Equation (8), the tangential circle radius *r* and base circle radius $d_{b}$ can be written as:

(10) $$d_{b}=\frac{4R\times \sqrt{{\left(x_{1}-x_{0}\right)}^{2}+{\left(y_{1}-y_{0}\right)}^{2}}-\left(d_{\max}^{2}-d_{\min}^{2}\right)}{2\left(d_{\max}-d_{\min}\right)}$$

(11) $$r=\sqrt{{\left(R-\sqrt{{\left(x_{1}-x_{0}\right)}^{2}+{\left(y_{1}-y_{0}\right)}^{2}}\right)}^{2}-{\left(d_{b}+d_{\min}\right)}^{2}}$$

**Figure 4:**
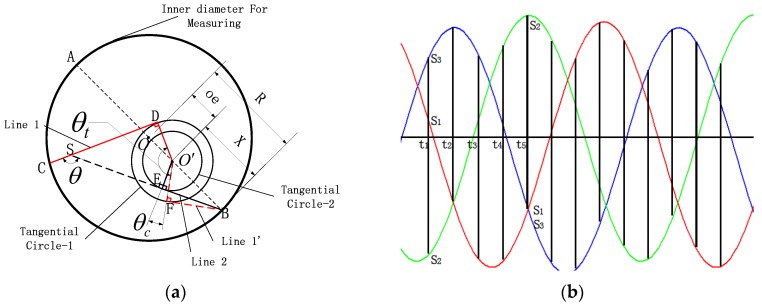
(**a**) Calibration principle of beam angle θ; (**b**) Sensor output waveform.

The calibration method of the angle between two adjacent beams is performed in the second phase of the calibration. The calibration principle and three sensors’ output waveforms are shown in Figure 4. 

The parameters $d_{b}$, $\theta_{c}$ and r are the natural parameters of the measuring device and they are not affected by the hole to be measured. Thus, the calibration process can use a standard ring gauge with known radius to get the parameters of the measuring device. The calibration principle of beam angle is shown in Figure 4a, in Status 1: Line 1 and Line 2 denote two adjacent laser beams, Line 1 directed point *C*, Line 2 directed point *B* where it obtained minimum measured value; in Status 2: Line 1 rotated anticlockwise to Line $1^{\prime }$ and point at point *B* obtains minimum measured value too. From Status 1 to Status 2 Line 1 rotated at an angle of $\theta_{t}$, but in Status 1 the angle between Line 1 and Line 2 is θ, because the point *B* is the feature point. Thus, due to the rotation of the measurement device, Line 1 pointed to the point *B* in Status 2 as the Line 1’ shown in the figure. If Line 1 and Line 2 point at point *B* at the same time, the angle between tangential circle-1 radius $r_{1}$ and tangential circle-2 radius $r_{2}$ is $\theta_{c}$. As shown in the Figure 4a, in the quadrilateral $SDO^{\prime }E$, $∠{\mathrm{DO}}^{\prime }E=\theta$, $∠{\mathrm{EO}}^{\prime }F=\theta_{c}$ and $∠{\mathrm{DO}}^{\prime }F=\theta_{t}$, and $∠{\mathrm{DO}}^{\prime }E=-∠{\mathrm{EO}}^{\prime }F+∠{\mathrm{DO}}^{\prime }F$. However, the positive and negative of $\theta_{c}$ is different in different status. Thus, as shown in the figure, the value of θ can be written as

(12) $$\theta =\theta_{c}+\theta_{t}$$

The point *B* where the sensor obtains minimum measured value is called feature point. When the laser beams direct at the feature point and get the minimum measured value, point *B*, point $O^{\prime }$ and point O are on the same line, because *X* is the distance between feature point *B* and the laser beam’s rotation center $O^{\prime }$, so *X* can be written as:

(13) X=R−oe

In the calibration process where the *R* can be acquired by standard ring gauge, the value of *oe* comes from Equation (9). As shown in Figure 4, in the calibration process, the angle $\theta_{c}$ between Lines 1 and 2 can be written as the equation below:

$$\theta_{c}=f\left(r_{1},r_{2},X\right)$$

**Figure 5:**
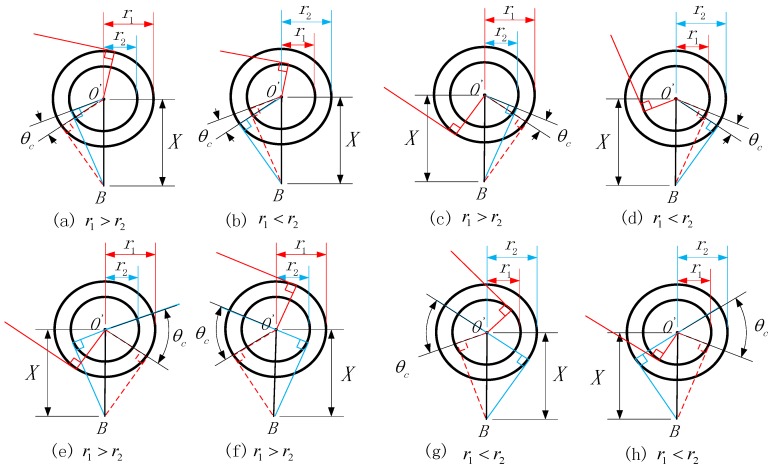
Different status of the calibration of $\theta_{c}$.

When the laser beam towards the feature point, it is tangent to the tangential circle. However, there are also two straight lines tangent to a circle by a point outside the circle. In the calibration process, there two laser beams and, combined, the position relation between two laser beams and the size relation between $r_{1}$ and $r_{2}$, the calibration of $\theta_{c}$ has eight different conditions, which are shown in Figure 5.

As shown in Figure 5, in Figure 5a $r_{1}>r_{2}$, and in Figure 5b $r_{1}<r_{2}$. In these two figures $\theta_{c}$ can be written as $\sin^{-1}\left(\frac{r_{1}}{X}\right)-\sin^{-1}\left(\frac{r_{2}}{X}\right)$ so we can find that the size relation between $r_{1}$ and $r_{2}$ has no effect on $\theta_{c}$. Taking the line connecting the feature point and the rotation center as the reference line and the rotation direction as the vector, the laser beam on the side of the vector head is called “advanced” and the laser beam on the vector tail is called “backward”, so the laser beam in Figure 4 are all at “backward” status and the laser beams in Figure 5c are all at “advanced” state. According the “advanced” and “backward” status we can acquire the value of $\theta_{c}$. Through the other figures in Figure 5, the conclusion is that $\theta_{c}$ is determined by the value of $r_{1}$, $r_{2}$ and *X* which has already been proposed. So, the $\theta_{c}$ can be expressed as Equation (14):

(14) $$\left\{ \begin{array}{l} \theta_{c}=+\sin^{-1}\left(\frac{r_{1}}{X}\right)-\sin^{-1}\left(\frac{r_{2}}{X}\right)(\mathrm{Line}1,\mathrm{Line}1,\mathrm{backward})\\ \theta_{c}=-\sin^{-1}\left(\frac{r_{1}}{X}\right)+\sin^{-1}\left(\frac{r_{2}}{X}\right)(\mathrm{Line}1,\mathrm{Line}1,\mathrm{advanced})\\ \theta_{c}=-\sin^{-1}\left(\frac{r_{1}}{X}\right)-\sin^{-1}\left(\frac{r_{2}}{X}\right)(\mathrm{Line}1,\mathrm{advanced},\mathrm{Line}2,\mathrm{backward})\\ \theta_{c}=+\sin^{-1}\left(\frac{r_{1}}{X}\right)+\sin^{-1}\left(\frac{r_{2}}{X}\right)(\mathrm{Line}1,\mathrm{backward},\mathrm{Line}2,\mathrm{advanced}) \end{array}\right.$$

“Backward” and “advanced” status is determined by the number of sensor sampling points. In counterclockwise rotation status, when the laser beam is in backward status the rotation angle from the minimum point to the maximum point in the rotation direction is less than π, and the angle from the maximum point to the minimum point is larger than π. Based on this conclusion, the number of sampling points between the minimum value and the maximum value is *n*, and the number of sampling points between the maximum value and the minimum value of the laser beam is *m*. If *n* is less than m this laser beam is in “backward” status, if it is the inverse, it is in “advanced” status.

Figure 4b shows the output waveforms of three laser sensors (different colour represents different sensor); every sensor gets a maximum measuring value and a maximum measuring value for each revolution. As shown in Figure 4b, at the $t_{2}$ time point the sensor-3 (blue) acquired maximum measuring value and at $t_{5}$ time point sensor-2 (green) acquired maximum measuring value. The inner measuring device was equipped with the machine tool spindle and its rotating speed is ω, so the rotation angle form $t_{2}$ to $t_{5}$ can be written as:

(15) $$\theta_{t}=\Delta t\times \omega$$

Equations (14) and (15) are taken into Equation (13) to calibrate the angle.

**Figure 6:**
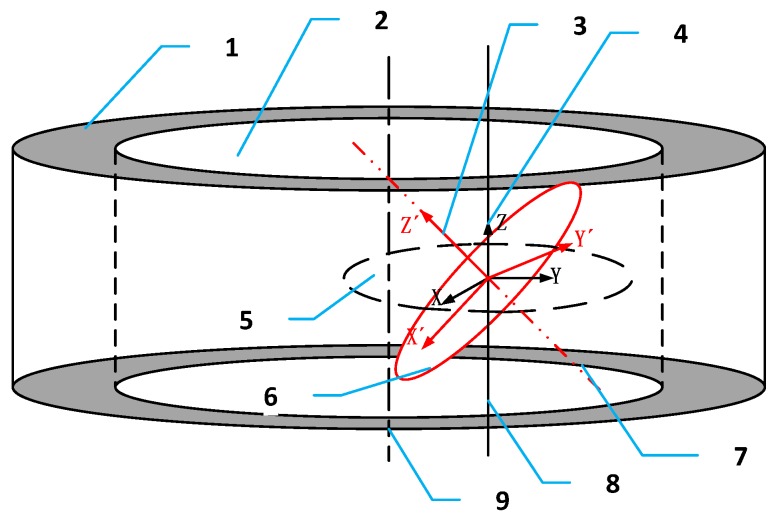
1. Measured workpiece; 2. Inner hole; 3. Ideal coordinate system; 4. Real coordinate system; 5. Ideal coordinate plane; 6. Actual coordinate plane; 7. Rotation axis; 8. Ideal axis; 9. Axis of measured hole.

The entire calibration principle above is based on an important prerequisite, which is that the rotation axis of the inner diameter measuring device is parallel to the axis of the measured hole and the laser beams in the inner diameter measuring device are on the same plane, but in fact this prerequisite is very difficult to achieve in calibration process. Thus, it is necessary to analyze how real conditions influence the calibration process. Figure 6 shows real status and ideal status of inner diameter measuring device. Obviously the rotation axis of inner diameter measuring device has an angle to the ideal axis, and the ideal axis is parallel to the axis of measured hole.

The real coordinate system can be acquired through the ideal coordinate system by the means of a rotation matrix [27]. In Figure 6, the ideal coordinate system rotates about the *y*-axis first then rotates about the *x*-axis. The rotation angles are $\theta_{y}$ and $\theta_{x}$, so the point P(x,y,z) under the ideal coordinate system can be written as $P^{\prime }\left(x^{\prime },y^{\prime },z^{\prime }\right)$ under the real coordinate system and Equation (16) establishes the relationship between point P and $P^{\prime }$.

(16) $$\left[\begin{array}{l} x^{\prime }\\ y^{\prime }\\ z^{\prime } \end{array}\right]=\left[\begin{array}{ccc} \cos\theta_{y} & 0 & -\sin\theta_{y}\\ 0 & 1 & 0\\ \sin\theta_{y} & 0 & \cos\theta_{y} \end{array}\right]\left[\begin{array}{ccc} 1 & 0 & 0\\ 0 & \cos\theta_{x} & \sin\theta_{x}\\ 0 & -\sin\theta_{x} & \cos\theta_{x} \end{array}\right]\left[\begin{array}{l} x\\ y\\ z \end{array}\right]$$

According to Equation (16), $x^{\prime }$, $y^{\prime }$, $z^{\prime }$ can be written as Equation (17):

(17) $$\left\{ \begin{array}{l} x^{\prime }=f_{x}\left(\theta_{x},\theta_{y},x,y,z\right)\\ y^{\prime }=f_{y}\left(\theta_{x},\theta_{y},x,y,z\right)\\ z^{\prime }=f_{z}\left(\theta_{x},\theta_{y},x,y,z\right) \end{array}\right.$$

In calibration process and measuring process laser displacement sensor’s output is the distance between two points, so under the ideal coordinate system the sensor’s output can be written as:

(18) $$d=\sqrt{{\left(x_{1}-x_{2}\right)}^{2}+{\left(y_{1}-y_{2}\right)}^{2}+{\left(z_{1}-z_{2}\right)}^{2}}$$

In Equation (18) the coordinate of $P_{1}$ is $\left(x_{1},y_{1},z_{1}\right)$ and $P_{2}$ is $\left(x_{2},y_{2},z_{2}\right)$. Taking them into Equation (17) can obtain $P_{1}^{\prime }\left(x_{1}^{\prime },y_{1}^{\prime },z_{1}^{\prime }\right)$ and $P_{2}^{\prime }\left(x_{2}^{\prime },y_{2}^{\prime },z_{2}^{\prime }\right)$. Then, taking $P_{1}^{\prime }\left(x_{1}^{\prime },y_{1}^{\prime },z_{1}^{\prime }\right)$ and $P_{2}^{\prime }\left(x_{2}^{\prime },y_{2}^{\prime },z_{2}^{\prime }\right)$ into Equation (18) can obtain $d^{\prime }$. Thus, $d^{\prime }$ can be written as:

(19) $$d^{\prime }=f_{d}\left(\theta_{x},\theta_{y},x_{1},x_{2},y_{1},y_{2},z_{1},z_{2}\right)$$

because the maximum measuring range of the leaser displacement sensor is 10 mm, so there is an Equation (20) under ideal coordinate:

(20) $$\left\{ \begin{array}{l} 10\leq \sqrt{{\left(x_{1}-x_{2}\right)}^{2}+{\left(y_{1}-y_{2}\right)}^{2}}\\ z_{1}=z_{2} \end{array}\right.$$

**Figure 7:**
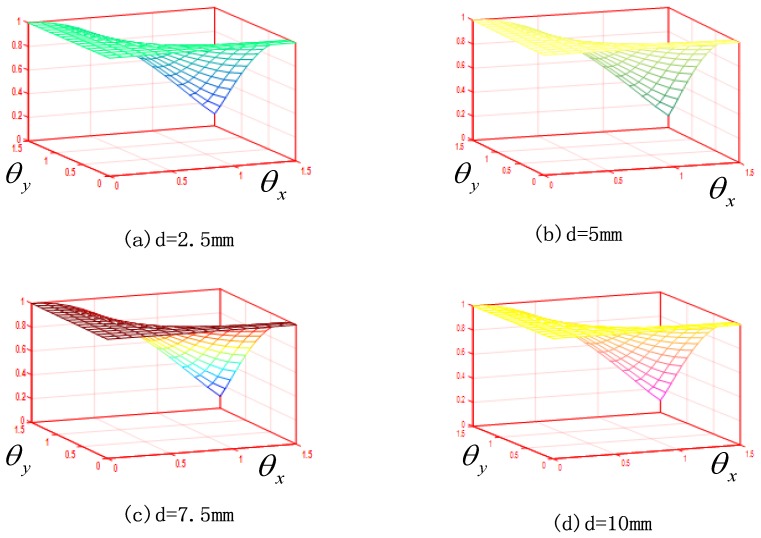
Effects of $\theta_{x}$ and $\theta_{y}$ on the value of $\frac{d}{d^{\prime }}$. (**a**) *d* = 2.5 mm; (**b**) *d* = 5.0 mm; (**c**) *d* = 7.5 mm; (**d**) *d* = 10.0 mm.

The value of $d^{\prime }$ is determined by $\theta_{x}$ and $\theta_{y}$ according to the Equation (20) if the coordinate of $P_{1}$ and $P_{2}$ are definite. Figure 7 displays the value of $\frac{d}{d^{\prime }}$ in different conditions of d. As shown in Figure 7, although the value of d is different, the value of $\frac{d}{d^{\prime }}$ has no significant change. According the equations above and Figure 7 some conclusions can be drawn: (1) the value of $\frac{d}{d^{\prime }}$ is not effected by the value of d but is effected by $\theta_{x}$ and $\theta_{y}$; (2) when $\theta_{x}$ is less than 21.8° and $\theta_{y}$ is less than 22.3° the value of $\frac{d}{d^{\prime }}$ approximated to 0.9873, but when $\theta_{x}$ and $\theta_{y}$ exceeds 21.8 and 22.3° the value of $\frac{d}{d^{\prime }}$ falls very fast. It is obvious that during the calibration and measurement process the value of $\theta_{x}$ and $\theta_{y}$ are far less than 21.8° and 22.3° so the effluence of $\theta_{x}$ and $\theta_{y}$ cannot be considered into calibration principle.

## 3. Experiment

### 3.1. Calibration Experiment of Base Radius and Tangential Radius

**Figure 8:**
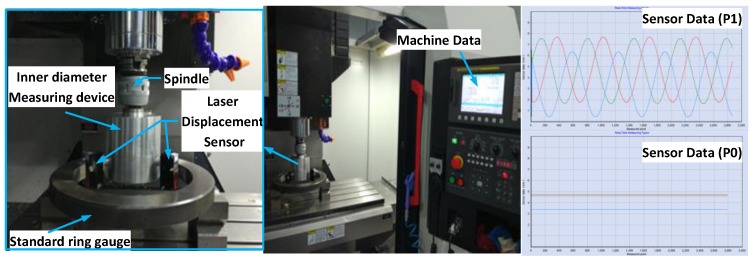
Experiment physical map.

According to the calibration principle above, the calibration process can be described as follows:

Step 1 Install the inner diameter measuring device on the spindle of the machine tool and adjust the spindle position so that the measuring device was placed on the ring gauge inner side whose inner diameter is 275.029 mm and maintained the sensors within the measuring range;
Step 2 Set the rotating speed of machine tool spindle at 60 r/min; because the position accuracy of machine spindle is 0.002 mm, the spindle mechanical coordinate *P*1 was recorded as (*x* = 262.955, *y* = −275.502);
Step 3 The machine tool rotated the inner diameter measuring device; each sensor got minimum value $d_{\min}$ and maximum value $d_{\max}$ during this process. Record the maximum and minimum values of every sensor for each revolution;
Step 4 After completing the multi-circles data acquisition at *P*1, the spindle was adjusted to the center *P*0 of the ring gauge by using the hole center positioning method mentioned above and the coordinates of point *P*0 is recorded as (*x* = 262.833, *y* = −273.182). As shown in Figure 8 the sensors’ output waveform was a straight line when the spindle was moved to point *P*0 [26];
Step 5 Take the coordinates of point *P*1 and point *P*0 into Equation (9) so the value of *oe* is 2.3222 mm. Calculate the average maximum value $\bar{d_{\max}}$ and average minimum value $\bar{d_{\min}}$ of each sensor, then take them into Equations (10) and (11) to get base circle radius $d_{b}$ and tangential circle radius *r*.

The experimental process is shown in Figure 8.

**Figure 9:**
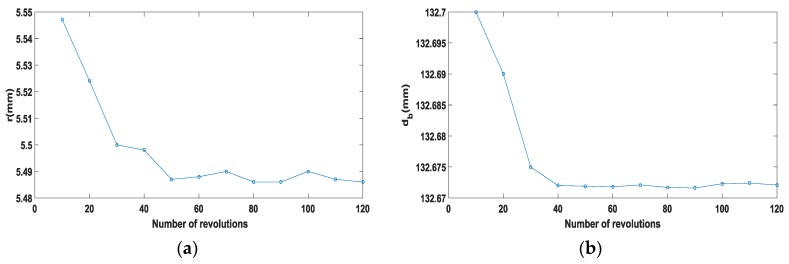
(**a**) The number of revolutions and *r*; (**b**) The number of revolutions and $d_{b}$.

Because the inner diameter measuring device is in a rotating status in the calibration process, the number of revolutions has an influence on the calibration result. To clarify the influence, the number of revolutions and calibration results is shown in Figure 9. Taking sensor-1 as an example, in Figure 9a the value of *r* (radius tangential circles radius) reached the minimum when the revolutions are 50 and when the revolutions exceed 50 the change of *r* tends to be gentle. In Figure 9b, when the number of revolutions exceed 40, the value changes of $d_{b}$ (base circle radius) tend to be gentle. According to Figure 9, we can find that if the numbers of revolutions exceed 50 the value of *r* and $d_{b}$ tend to gentle. On account of the fact that the rotating speed of machine tool spindle is 60 r/min, the period of calibration should exceed one minute or continue one minute at last. 

In accordance with the principle above, the result of calibration of three sensors can be acquired and are shown in Table 1.

### 3.2. Calibration Experiment of Laser Beams’ Angle

In Figure 4b, the laser displacement sensor’s output waveform is similar to the sine curve because in the calibration process the sensor is in the rotational scanning status. Therefore, the number of the sensor sampling points per circle has to be set before calibration. There is a fence effect in the sample of the sensor output data and the fence effect may cause serious errors in some conditions [28]. In this paper, how to set the number of sampling points per circle is based on the theory of fence effect [29]. Set *N* is the sampling point per circle, sampling interval α=2π/N, maximum angle error is $\sigma_{\alpha }$ = π/N, and the raw error of sensor is $\sigma_{d}=d/\left(\frac{1}{\cos\sigma_{\alpha }}-1\right)$, and *d* is the theoretical distance from the sensor to the measured point. In this paper, *d* is 147.546 mm. If the number of sampling points is more than 583 the error of the sensor is less than 0.4 μm, and if the number is more than 1000 the error will be less than 0.28 μm. But as the inner diameter measuring device uses wireless mean to send data, so the number of sampling points cannot shrink indefinitely. According to all the above the number of sampling points per circle is 625.

It is necessary to determine the “advanced” and “backward” status of the laser beams before calibrating the angle. Because the sensors are in the same time interval, the sampling status during the rotation scanning process and three sensors begin to sample at the same time. The number of sampling points every sensor is fixed at 625 and the rotation angular speed of the machine tool spindle is known so that the time interval between adjacent sampling points can be calculated. Therefore, it is only to know the quantity of sample points between two sampling moments that the interval time between two moments can be obtained. When the spindle rotates at the *P1* position, the serial number of sampling points of each sensor is recorded when it reaches the extreme point (maximum value $d_{\max}$ point, minimum value $d_{\min}$ point), and the difference between two adjacent extreme points sample serial number calculated as the quantity of sample points between two adjacent extreme points.

In Table 2, the first column is the serial number of sampling points when the sensor-1 is directed at minimum value point. The third column is the serial number of sampling points when the sensor-1 is directed at maximum value point. The second column and the fourth column is the difference of the serial number between the first column and the third column. For example, when the sensor-1 is first directed at the minimum value point, its sampling point serial number is 312, then it is directed at the first maximum value point and its sampling point serial number is 639, so the difference between 639 and 312 is 327. As shown in Table 2, 332 is the difference of the sampling point serial number between first maximum value point (639) and second minimum value point (971). In the last row, the table shown the mean difference between the sampling points of two extreme points for every sensor. 

As shown in Table 2, the first extreme simple point of sensor-1 and sensor-2 is the minimum value point, and the first extreme simple point of sensor-3 is the maximum value point. For the sensor-1, the mean difference 328 between the minimum value point and maximum value point is less than the mean difference 332 between the maximum value point and minimum value point, so the sensor-1 is at the “backward” status. Similarly, we can see the sensor-2 is at the “backward” status, sensor-3 is at the “advanced” status. According to Equation (14) the calibration of $\theta_{c}$ can be accomplished. 

**Figure 10:**
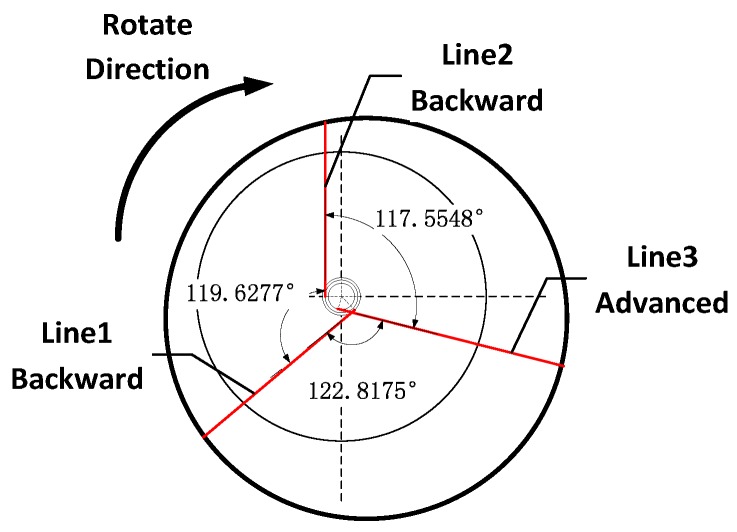
The angle between two adjacent laser beam.

According to the calibration method described in Equation (15), each sensor’s minimum value point is selected as its feature point. From Table 2, three sensors in the order of 2-1-3 directed at the feature point. Sensor-2 first directed at the feature point, then sensor-1 directed at the feature point; the mean difference of the number between two feature points is 222 (the mean difference of the number between the fifth column and second column). Similarly, the mean difference between sensor-1 and sensor-3 is 220 (the mean difference of the number between the ninth column and first column), and the mean difference between sensor-3 and sensor-2 is 219 (the mean difference of the number between the fifth column and ninth column). Finally, the calibration of $\theta_{t}$ can be accomplished and the result of the calibration of angle θ is shown in Figure 10.

### 3.3. Verification Experiment

**Figure 11:**
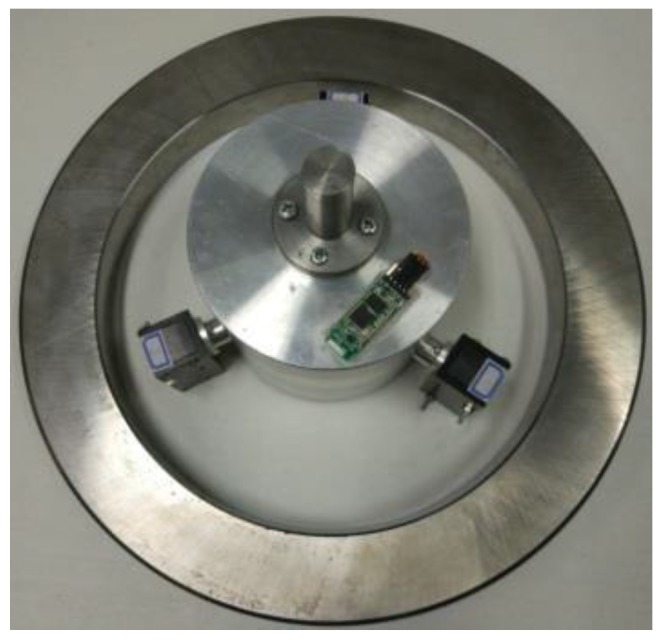
Verification experiment.

In order to test the effectiveness of the calibration result and inner diameter measuring device, take the tangential circles radius (r), base circles radius ($d_{b}$) and angles (θ) between two adjacent laser beams into Equations (3)–(7), and put the measuring device into the standard ring gauge as shown in Figure 11 to measure the diameter of standard ring gauge according the actual mathematical model. In order to ensure the credibility of the experiment we repeated 10 experiments and each time the inner diameter measuring device was moved to a new position. The results of multiple measurements is shown in the Table 3.

It can be seen from the experiment results that the measurement error of the inner diameter measuring device was below ±15 μm and the relative error was less than 0.011% with the new mathematical model. Comparing with the uncalibrated mathematical model discussed in the previous chapter, the measurement accuracy has been improved to some extent and this method has certain practical significance.

## 4. Sensor Raw Error

**Figure 12:**
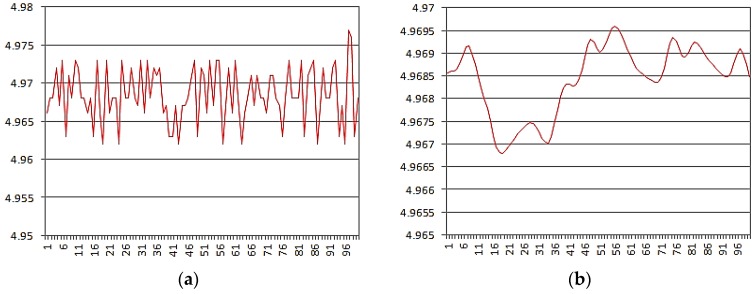
(**a**) Raw data; (**b**) Filtered data.

The laser displacement sensor used in the experiment is a Panasonic HG-C1030; its measuring range is ±5 mm, the repeatability is 10 μm, the linearity is ±0.1% FS, and the reaction time can be switched within 1.5 ms, 5 ms or 10 ms. The output is analog signal. As the sensor output data needs to go through through AD conversion so that can be sent to the host computer for processing, so the sensor raw error and AD conversion can cause data beating. Therefore, in order to stabilize measurement results, median filter, average filter and kalman filter was used to filter sensor raw data [30]. The sensors raw data and filtered data are shown in Figure 12.

As shown in Figure 12, the value of the data beat is 15 μm and the mean value of the measurement was 4.9685 mm before filtering, and the value of beat data reduced to 3 μm and the mean value was 4.9684 mm after filtering. Obviously, the stability of the data is significantly improved after filtering, and its measurement results has more reference value.

In order to further assess the sensor measurement data, it is necessary to use a sensor whose measurement accuracy is higher than the laser displacement sensor. So, we used the high-precision ID-0530 Mitutoyo digital micrometer (indication error of ±1.5 μm) and laser displacement sensor to measure the same amount of displacement simultaneously, and then assessed the laser sensor measurement accuracy. The experimental result is shown in Table 4. In the process of this experiment we collected 12 sets of data continuously. Table 4 reflects the effect of two sensors measuring the same displacement. Relative to the Mitutoyo digital micrometer, the laser displacement sensor measurement error is about ±9 μm, in line with the sensor nominal value, in other words the filtering process can not only reduce the data fluctuations, but also can reduce the sensor measurement error, making the sensor measurement accuracy increase to the original level of the sensor without AD conversion.

## 5. Conclusions

Based on the inner diameter measuring device and the measurement method proposed by the research group, a mathematical model of the actual model of the inner diameter measuring device is proposed. After calibration, the inner diameter measuring device obtained the diameter of the standard ring gauge by an actual model. The advantages of the calibration method proposed in this paper are summarized as follow: (1) This calibration method improved the measurement accuracy and measurement range of the inner diameter measuring device with only three sensors; (2) The calibration method proposed in this paper solves the problem of how to measure the angle between two adjacent laser beams; (3) The inner diameter measuring device has the ability to automatically measure and in situ measure after calibration; (4) The inner diameter measuring device can measure inner diameters bigger than 250 mm with the measurement uncertainty of 15 μm; (5) The internal parameters of the measuring device are obtained after calibration, so in the measuring process it is not necessary to be installed on the spindle of the machine tool and the error of the machine tool can be ignored. In other words, the inner diameter measuring device achieved static measurement without rotation; (6) The calibration method and measurement method in this paper are not only applicable to the hole whose diameter is larger than 250 mm, but also applicable to holes with significantly larger diameter if the measurement device is suitable.

## Figures and Tables

**Table 1:** Base radius and tangential radius (mm).

	Sensor-1	Sensor-2	Snesor-3
$\bar{d_{max}}$	7.0549	6.9256	5.7177
$\bar{d_{min}}$	2.4067	2.2804	1.073
r	5.5484	2.5295	1.5262
$d_{b}$	132.6718	132.8883	134.1108

**Table 2:** Sensor extreme point sequence number.

Sensor-1				Sensor-2				Sensor-3			
$d_{\min}$		$d_{\max}$		$d_{\min}$		$d_{\max}$		$d_{\min}$		$d_{\max}$	
Number	Difference	Number	Difference	Number	Difference	Number	Difference	Number	Difference	Number	Difference
312	327	639	332	88	330	418	331	531	332	199	327
971	329	1300	328	749	330	1079	329	1188	330	858	330
1628	329	1957	330	1408	330	1738	329	1847	329	1518	333
2287	329	2616	333	2067	332	2399	330	2511	331	2180	327
2949	326	3275	335	2729	327	3056	330	3170	332	2838	328
3610	327	3937	332	3386	331	3717	329	3831	333	3498	325
4269	326	4595	331	4046	331	4377	330	4489	333	4156	329
4926	330	5256	333	4707	329	5036	331	5148	330	4818	327
5589	327	5916	330	5367	327	5694	334	5808	333	5475	328
6246	331	6577	332	6028	328	6356	330	6468	332	6136	329
6909	328	7237	328	6686	329	7015	330	7128	331	6797	327
7565	329	7894	335	7345	330	7675	328	7788	333	7455	327
8229	325	8554	333	8003	333	8336	327	8446	331	8115	330
8887	328	9215	330	8663	332	8995	331	9106	330	8776	329
9545	330	9875	331	9326	326	9652	333	9766	331	9435	329
10,206	328	10,534	332	9985	326	10,311	334	10,420	325	10,095	335
10,866	326	11,192	336	10,645	329	10,974	331	11,085	330	10,755	331
11,528	327	11,855		11,305	330	11,635		11,746	330	11,416	
	**Mean 328**		**Mean 332**		**Mean 329**		**Mean 331**		**Mean 331**		**Mean 329**

**Table 3:** Experiment data of inner diameter measurement (mm).

	Value of Sensor-1	Value of Sensor-2	Value of Sensor-3	Diameter	Error
No.1	2.8743	6.3246	3.4249	275.021	−0.0083
No.2	3.7568	1.5689	7.4925	275.039	0.0100
No.3	4.2155	3.5671	4.9925	275.028	−0.0008
No.4	5.3617	2.8483	4.5986	275.036	0.0070
No.5	6.8716	4.6248	1.1909	275.044	0.0146
No.6	7.3256	2.7812	2.6612	275.036	0.0073
No.7	8.5649	3.2114	0.9164	275.043	0.0139
No.8	3.5188	5.2147	3.9581	275.022	−0.0075
No.9	2.3544	8.5528	1.5436	275.023	−0.0062
No.10	4.0126	3.5167	5.2671	275.042	0.0131

**Table 4:** Experimental of actual measurement parameters of sensors (mm).

	Laser Sensor	Laser Sensor	ID-0530	Error
	Filter Data	Displacement	Displacement	
No. 1	0.0380		0	
No. 2	0.9542	0.9162	0.9070	−0.0092
No. 3	1.9071	1.8691	1.8655	−0.0036
No. 4	2.9721	2.9341	2.9250	−0.0091
No. 5	3.9751	3.9371	3.9375	0.0004
No. 6	4.9684	4.9304	4.9245	−0.0059
No. 8	6.0211	5.9831	5.9850	0.0019
No. 9	7.0031	6.9651	6.9610	−0.0041
No. 10	7.9963	7.9583	7.9595	0.0012
No. 11	8.9896	8.9516	8.9615	0.0099
No. 12	9.9365	9.8985	9.9015	0.0030
